# The effect of fatigue on asymmetry between lower limbs in functional performances in elite child taekwondo athletes

**DOI:** 10.1186/s13018-020-02175-7

**Published:** 2021-01-09

**Authors:** Yanfei Guan, Shannon Bredin, Qinxian Jiang, Jack Taunton, Yongfeng Li, Nana Wu, Lina Wu, Darren Warburton

**Affiliations:** 1 School of Kinesiology, University of British Columbia, 2259 Lower Mall Research Station, Vancouver, V6T 1Z4 Canada; 2 Department of Physical Education, Weifang Medical University, Weifang, China; 3 Allan McGavin Sport Medicine Center, University of British Columbia, Vancouver, Canada; 4 College of Sports and Health, Shandong Sport University, Ji’nan, China; 5 School of Nursing and Health, Qingdao Huanghai University, Qingdao, China

**Keywords:** Bilateral asymmetry, Fatigue, Injury, Lower-limb power, Dynamic balance, Muscle flexibility

## Abstract

**Background:**

Inter-limb asymmetry above a certain threshold in functional performance indicates increased injury risk in sports. Fatigue has been found to increase bilateral asymmetry in lower-limb jumping performance among high-school and adult athletes, whereas this impact has not been examined in child athletes. This study aimed to examine the effect of fatigue on inter-limb asymmetry in functional performances in elite Taekwondo athletes aged between 9 and 11 years.

**Methods:**

Performance of single-leg jumps, Star Excursion Balance Test (SEBT), and muscle (hamstring and gastrocnemius) flexibility were measured for 13 elite male child Taekwondo athletes (aged 9.85 ± 0.80 years) at both the rested and fatigued states to examine the inter-limb asymmetry. A two-way repeated measures ANOVA was conducted to examine for difference and the interaction between limb (dominant, non-dominant leg) and state (rested, fatigued state) for each test. Paired *t* test or Wilcoxon signed-rank test was used to compare the asymmetry magnitude at the rested vs. fatigued state for each test, and the variation of performance post fatigue in the dominant vs. non-dominant leg when appropriate.

**Results:**

The inter-limb asymmetry in triple-hop distance significantly (*p* = 0.046) increased with fatigue, whereas the asymmetry significantly (*p* = 0.004) decreased with fatigue in anterior (ANT) reach distance in SEBT. A significant (*p* = 0.027) limb by state interaction was shown for posterolateral (PL) reach distance in SEBT, wherein a significant (*p* = 0.005) bilateral difference was only shown at the rested state. The PL reach distance showed a significantly greater decrease (*p* = 0.028) post fatigue when using the dominant leg for support compared to using the non-dominant leg.

**Conclusions:**

Fatigue significantly impacts inter-limb asymmetry in jump performances and dynamic balance for child athletes, while the variation of inter-limb asymmetry post fatigue may be different across tests. For the purpose of injury prevention, practitioners should consider assessing the inter-limb asymmetry for children at both the rested and fatigued state and be mindful of the fatigue response of each leg in functional tests.

## Introduction

Inter-limb asymmetry (or bilateral difference, bilateral asymmetry) is defined as differences in the function or performance between the dominant and non-dominant limb [1]. Inter-limb asymmetry may emerge from long-term training in the same sport [2, 3]. There is a growing interest on the topic of inter-limb asymmetry because of its influence on risk for sport injury. The inter-limb asymmetry may potentially place the lower extremities of both sides at a heightened risk of sport injury [4]. The strong leg may sustain overloading of muscle-tendon components because of increased dependence and prolonged exposure to high force in sport activities, while it may be difficult for the weak leg to manage even average stress and force [4]. The bilateral asymmetry in lower-limb strength and power [5], dynamic balance [6, 7], and muscle flexibility [5] have been associated with increased injury risk in high-school and collegiate athletes.

Current literature has suggested the importance of investigating the impact of fatigue on inter-limb asymmetry, due to the potential for asymmetry becoming more prominent with fatigue [8]. The increased inter-limb asymmetry may play a role in the heightened injury risks under the fatigued state [9]. A number of studies have focused on the running biomechanics with inconsistent findings. Radzak et al. [10] reported that fatigue amplified the inter-limb asymmetry in knee internal rotation and knee stiffness (increased by 14% and 5.3%, respectively) in running (4 m/s) movement of the healthy adults, whereas the inter-limb asymmetry in vertical stiffness and loading rate decreased post fatigue. However, most of the previous studies revealed that there was no impact of fatigue on kinematics and kinetics in running movement [11–14].

In contrast, there is a paucity of research examining the acute impact of fatigue on inter-limb asymmetry in functional performances. The unilateral jump tests have been widely used for assessing the inter-limb asymmetry in lower-limb power as the single-leg jumping movements are common in sports and the assessment is time-efficient [9]. Previous findings showed that the inter-limb asymmetry in single-leg countermovement jump (CMJ) performance increased post fatigue among adolescents and adults [9, 15, 16]. In addition, the Star Excursion Balance Test (SEBT) and its modified version (Y Balance Test) have been widely used to examine the inter-limb asymmetry in dynamic balance and neuromuscular control. However, to the best of our knowledge, the acute effect of fatigue on inter-limb asymmetry in SEBT or Y Balance Test performance has not been reported. The influence of fatigue on inter-limb asymmetry in muscle flexibility which has been reported as a risk factor for sport injury [5] is also not clear.

Current literature has mostly examined athletes in high-school and adulthood years, whereas there is a paucity of research focused on child athletes. Compared to adult athletes, athletes in childhood-age are more vulnerable to injuries because the immature cartilage and muscles are more susceptible to injuries in sports [17, 18]. Acute and overuse injuries in the growth cartilage may cause permanent damage to bone growth if they were not treated well [18]. Radelet et al. [18] have reported that the injury rate ranged from 1.0 to 2.3 per 100 athlete exposures in 7–13-year-old children in community sports. Examining the inter-limb asymmetry in functional performances and the impact of fatigue on this asymmetry is important for the purpose of injury prevention among child athletes, due to the association between inter-limb asymmetry and injury [5–7]. Therefore, the purpose of the present investigation was to examine the acute impact of fatigue on inter-limb asymmetry in lower-limb power, dynamic balance, and muscle flexibility among child athletes. The present study fits into the framework of translational orthopedics by filling the gap between basic sciences and clinical sciences [19, 20]. The potential findings may provide reliable methods (single-leg jump tests, SEBT, and flexibility tests) for practitioners to evaluate the effect of fatigue on inter-limb asymmetry in functional performances in child athletes. In addition, generating these findings may contribute to understanding the association between fatigue, inter-limb asymmetry, and injury, which may contribute to injury prevention and injury prediction in clinical practice.

## Methods

### Participants

A total of 13 elite male Taekwondo athletes (height = 144.31 ± 7.81 cm, body mass = 37.58 ± 9.20 kg, age = 9.85 ± 0.80 years, training years = 3.31 ± 0.86 years) between 9 and 11 years of age were recruited. All participants had accepted single-sport training (specialized at Taekwondo) and won medals in national level or provincial tournaments. All participants had at least 1 year of training experience and maintained regular training (3-4 h per day, 4 days per week) in the preceding 12 months before participation.

### Assessments of bilateral asymmetry

#### Lower-limb power

Lower-limb power tests included the single-leg countermovement jump (CMJ), hop, and triple-hop tests. The goal of the single-leg CMJ test was to obtain the maximum jump height of each leg after performing a single-leg countermovement. Participants stood in an upright position with feet positioned shoulder width apart. The hands were placed on hips during the entire movement to reduce the impact of arm movement [21]. To start the test, the participant lifted one leg to a self-selected position, and then performed a countermovement followed by a vertical jump. The jump was recorded with an iPhone 6 s (Apple, Inc., USA) at 240 Hz. The jump height (in cm) was calculated based on the flight time of the jump by identifying the take-off and landing frames using the “My Jump” iPhone application, which has been reported a valid and reliable method [22]. Three valid trials were required for each leg. The jump height (in cm) for each trial was recorded. The average jump height of the three trials of each leg was used for analysis.

The goal of the single-leg hop and triple-hop test was to obtain the maximum horizontal distance of a hop and three consecutive hops, respectively. Participants started with a unilateral standing position and toes of the supporting leg behind the starting line. For the one hop test, participants were instructed to perform a forward hop as far as possible and land firmly with the same leg. Failure to perform a firm landing was viewed as an invalid trial. The hop distance (in cm) from the starting line to the participant’s landing heel was measured and recorded. For the triple-hop test, participants were instructed to perform three consecutive forward hops as far as possible using the same leg, with the intention of reducing the floor contact time of the first two landings as much as possible. The landing of the last hop had to be firm. Failure to perform a firm landing at the last hop was viewed as an invalid trial. The total hop distance (in cm) from the starting line to the participant’s final landing heel was measured and recorded. Three valid trials were required for each leg in each test. The average distance (in cm) of the three trials of each leg in each test was used for analysis.

#### Dynamic balance

**Fig. 1:**
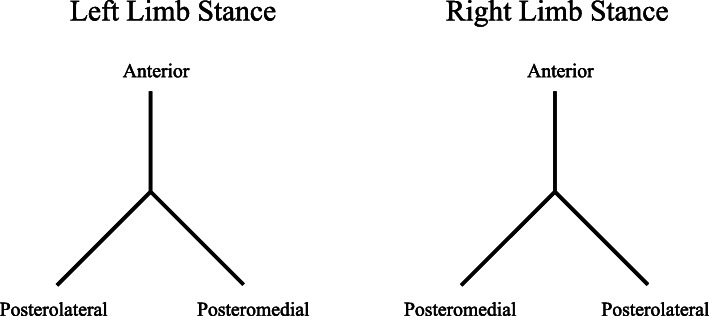
The sketch of the Star Excursion Balance Test

Dynamic balance was measured using a simplified version of the Star Excursion Balance Test (SEBT) which is a valid and reliable (ICC = 0.78–0.96) [23] test developed by Gray [24]. The sketch of the SEBT is presented in Fig. 1. The goal of this test was to obtain the maximum reach distance along three directions (anterior [ANT], posteromedial [PM], and posterolateral [PL]) using the contralateral leg while maintaining a unilateral stance with solid foundation [25]. While standing with a single leg at the convergence of reach direction lines, participants were required to reach as far as possible with the other leg along each of the three directions (in the order of ANT, PM, and PL direction), lightly touching each line using the most distal part of the reaching foot without disrupting the established balance during the entire movement. The point where the most distal part of the foot reached was marked with erasable ink on each direction line. Each participant performed three trials (each trial with three directions) using each leg. Participants took this test barefoot to eliminate the effects of shoes on balance and stability. The trial was viewed as invalid when participants failed to maintain the unilateral stance, moved or lifted the standing foot from the convergence of lines, or failed to return the reaching foot to the original position [6]. The greatest reach distance (in cm) from the convergence of lines to the point where the most distal part of the foot reached in three trials for each direction of each leg was measured and used for analysis [6].

#### Hamstring and gastrocnemius flexibility

**Fig. 2:**
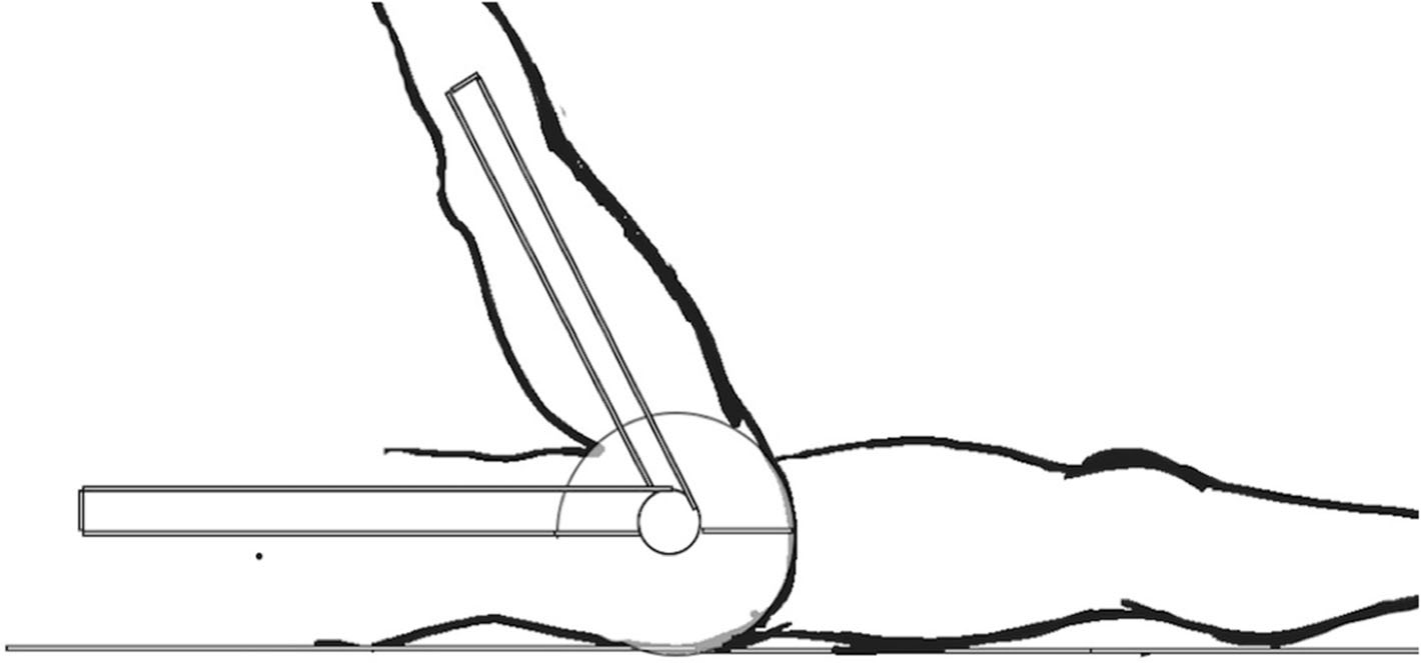
Goniometric measurement of flexibility in the hamstring muscles

**Fig. 3:**
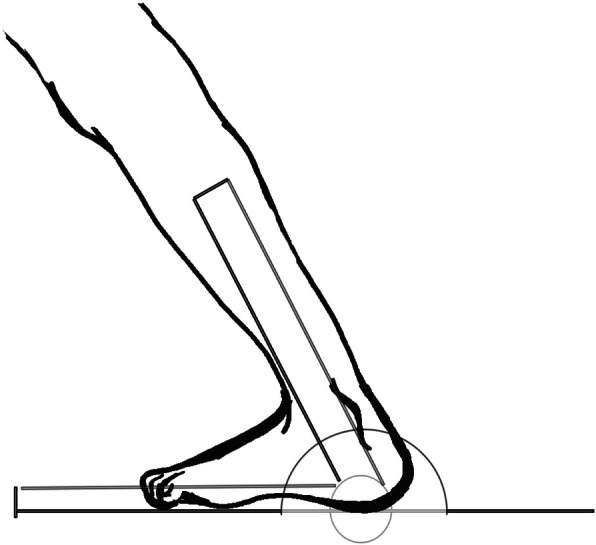
Goniometric measurement of flexibility in the gastrocnemius muscles

Hamstring and gastrocnemius flexibility was measured using a goniometer. Specific protocols for measurements are reported elsewhere [26]. The goal of this test was to obtain the maximum range of motion of hip and ankle joint in a specific position reflecting the flexibility of the hamstring and gastrocnemius. The flexibility of the hamstring was measured with the participant in a supine position on a table (Fig. 2). The participant lifted the tested leg and kept it straightening with the help from an examiner. The axis of the goniometer was placed at the great trochanter. The stationary arm of the goniometer was placed horizontally at the table, and the moving arm was placed pointing to the lateral epicondyle of the femur. The angle (°) of the maximum flexion at the hip joint was measured. The flexibility of the gastrocnemius was measured with the participant fully extending the knee of the tested leg and maximally flexing the ankle of the tested leg while maintaining the sole on the floor (Fig. 3). The stationary arm of the goniometer was placed horizontally to the floor, and the moving arm was placed pointing to the most distal part of the fibula. The angle (°) of the ankle dorsiflexion was measured. When a variation above 5% was found between two trials of each flexibility test, an extra trial was performed. The two most closely related values were recorded and used for analysis.

### Fatigue induction

Before performing the fatigue protocol, the participant was asked to perform a broad jump (three trials) to obtain the maximum horizontal distance (in cm) achieved at a rested state. The fatigue protocol was modified from previous research [27, 28]. First, the participant performed two sets of 30-s consecutive double chop kicks on the punching bag at the maximum frequency, with a break of 30 s between sets. After completing the kicks, the participant received a 30-s rest, followed by performing consecutive frog jumps until volitional exhaustion. This is one set of fatigue protocol. After one set of fatigue protocol, the participant was asked to test the broad jump again for horizontal distance. The criterion for fatigue was reached when the participant did not attain 90% of his maximum jump (broad jump) distance for three consecutive trials [27, 29]. If the criterion for fatigue was not reached, the participant began another set of fatigue protocol until reaching the criterion for fatigue.

### Procedures

Participants finished all assessments in one visit. First, anthropometric measurements including body height (cm), leg length (cm), and body weight (kg) were taken. Leg length was measured from the anterior superior iliac spine to the most distal aspect of the medial malleolus with participants lying supine on a table. Limb dominance was examined by letting the participant kick a soccer ball, and the limb which the participant preferred for the ball kicking was defined as the dominant leg. In the session, participants then completed stretching and a 5-min jog to warm-up. Participants were then provided with test instructions and received time to practice until they were familiar with each assessment, which included tests measuring lower-limb power (single-leg CMJ, hop, and triple hop), dynamic balance (SEBT), and muscle flexibility (hamstrings and gastrocnemius). Following test familiarization, participants received a rest interval of 5 min.

Testing followed a three-phase approach: (1) testing at a rested state, (2) fatigue induction, and (3) testing at a fatigued state. Testing at a rested state was conducted in the following order: single-leg CMJ, hop, triple hop, SEBT, and flexibility of the hamstring and gastrocnemius, with a 1-min rest interval between each assessment. At the end of the first phase, participants received a 5-min rest period, followed by the fatigue induction phase until the fatigue criterion was reached. The third phase, testing at a fatigued state, was conducted exactly the same as testing at the rested state except that participants were asked to run continuously between trials with no rest provided between assessments. The starting leg was randomly selected to reduce the effect of order.

### Data analysis

Dependent variables included the single-leg CMJ height, hop distance, triple-hop distance, the reach distance (normalized to leg length) at each direction (ANT, PM, PL, and the composite score of the three directions [COM]) in SEBT, the angles measured in hamstring and gastrocnemius flexibility tests, the inter-limb asymmetry in performance at the rested and fatigued state for each test, and the variation of performance from the rested to fatigued state (fatigue rate) in each test for each leg. The mean of each dependent variable in lower-limb power tests (single-leg CMJ, hop, and triple hop) over the three trials was calculated for each participant. The reach distance in SEBT was normalized to leg length (leg length %), and the COM reach distance was calculated by averaging the reach distance of the three directions for each leg. The magnitude of inter-limb asymmetry for each measurement was quantified using an equation modified from previous studies [30, 31]: Asymmetry Index = (Stronger Limb − Weaker limb) × 2/(Stronger Limb + Weaker limb) × 100%. The variation of performance from the rested to fatigued state was calculated as a percentage for each measurement of each leg: (Rested − Fatigued) × 2/(Rested + Fatigued) × 100%.

### Statistical analysis

Descriptive data are shown as mean ± standard deviation (SD). The reliability of each measurement was examined using the intra-class correlation coefficient (ICC). Normal distribution and homoscedasticity assumption of the data were examined using the Kolmogorov-Smirnov and Levene’s tests, respectively. A two-way repeated measures ANOVA was conducted to examine for difference and the interaction between limb (dominant, non-dominant leg) and state (rested, fatigued state) for each test. Where significant differences were found between limbs or states, paired *t* tests were performed. To compare the inter-limb asymmetry between the rested and fatigued state in jump performance (unilateral CMJ, hop, and triple hop) and muscle flexibility (hamstring and gastrocnemius), a non-parametric test (Wilcoxon signed-rank test) was conducted as the data distribution was not normal. To compare the inter-limb asymmetry between the rested and fatigued state in SEBT performance (ANT, PM, PL, and COM reach distance), paired *t* tests were performed. The variation of performance from the rested to the fatigued state (fatigue rate) was compared between the dominant and non-dominant leg using a paired *t* test for each test. Effect size (ES) was reported using Cohen’s *d* for results in *t* tests [32], and using the correlation coefficient for results in Wilcoxon signed-rank tests (*r* = *Z*/ $\sqrt{n}$) [33]. Statistical significance was set a priori at *p* < 0.05. All data analysis was conducted using SPSS 23.

## Results

The results of ICC (Table 1) showed an excellent reliability of each measurement in the single-leg jump tests, SEBT, and muscle flexibility tests for each leg.

**Table 1:** The intra-class correlation coefficient of each measurement

			Rested state		Fatigued state	
			ICC	95% CI	ICC	95% CI
Jumps	CMJ	DL	0.788	0.467–0.929	0.824	0.556–0.942
		NDL	0.888	0.723–0.963	0.945	0.863–0.982
	Hop	DL	0.842	0.606–0.947	0.916	0.744–0.974
		NDL	0.884	0.704–0.962	0.959	0.887–0.987
	Triple Hop	DL	0.930	0.815–0.978	0.866	0.656–0.958
		NDL	0.934	0.830–0.979	0.938	0.835–0.980
SEBT	ANT RD	DL	0.990	0.970–0.997	0.994	0.973–0.998
		NDL	0.986	0.951–0.996	0.977	0.911–0.993
	PM RD	DL	0.993	0.947–0.998	0.956	0.870–0.986
		NDL	0.985	0.923–0.996	0.989	0.961–0.997
	PL RD	DL	0.993	0.959–0.998	0.995	0.986–0.998
		NDL	0.990	0.973–0.997	0.995	0.982–0.999
Flexibility	Hamstring	DL	0.997	0.950–0.999	0.990	0.959–0.997
		NDL	0.989	0.963–0.997	0.997	0.964–0.999
	Gastrocnemius	DL	0.991	0.971–0.997	0.988	0.955–0.996
		NDL	0.991	0.969–0.997	0.990	0.919–0.998

*ICC* intra-class correlation coefficient, *CI* confidence interval, *CMJ* single-leg countermovement jump, *SEBT* Star Excursion Balance Test, *RD* reach distance, *ANT* anterior, *PM* posteromedial, *PL* posterolateral, *DL* dominant leg, *NDL* non-dominant leg

### Lower-limb power

Descriptive statistics for lower-limb power tests are presented in Table 2. There was a significant main effect of state for single-leg CMJ height (*F*_(1,12)_ = 57.880, *p* = 0.000, *η*^2^ = 0.828), hop distance (*F*_(1,12)_ = 87.557, *p* = 0.000, *η*^2^ = 0.879), and triple-hop distance (*F*_(1,12)_ = 47.667, *p* = 0.000, *η*^2^ = 0.799). For each leg, participants showed significantly better performance at the rested state compared to that at the fatigued state in each test (*p* < 0.05, ES ≥ 1.148).

**Table 2:** Descriptive statistics for single-leg jump performances

Parameters	Limb	Rested state	Fatigued state
CMJ (cm)^a^	DL	14.31 ± 2.05*	12.77 ± 2.02
	NDL	13.95 ± 2.80*	12.29 ± 2.68
Hop (cm)^a^	DL	130.48 ± 12.68*	119.97 ± 12.71
	NDL	125.23 ± 15.06*	117.28 ± 17.58
Triple Hop (cm)^a^	DL	421.82 ± 31.61*	396.42 ± 32.02
	NDL	410.44 ± 39.42*	378.26 ± 44.78

*CMJ* single-leg countermovement jump, *DL* dominant leg, *NDL* non-dominant leg

*Significant difference between the rested and fatigued state

^a^Main effect of state

### Dynamic balance

Descriptive statistics for the SEBT are presented in Table 3. There was a significant main effect of state for ANT (*F*_(1,12)_ = 8.113, *p* = 0.015, η^2^ = 0.403), PM (*F*_(1,12)_ = 8.850, *p* = 0.012, *η*^2^ = 0.424), and COM (*F*_(1,12)_ = 4.997, *p* = 0.045, *η*^2^ = 0.294) reach distance. A significant (*F*_(1,12)_ = 6.312, *p* = 0.027, *η*^2^ = 0.345) limb by state interaction was shown for the PL direction: the reach distance at the rested state was significantly (*p* = 0.005, ES = 0.938) greater when establishing the unilateral stance using the dominant leg compared with using the non-dominant leg, while no significant (*p* > 0.05) difference was shown between the two sides at the fatigued state. Results of paired *t* tests showed that PM reach distance at the rested state was significantly greater (*p* = 0.023, ES = 0.722) than that at the fatigued state when establishing the unilateral stance using the dominant leg, whereas there was no significant difference (*p* > 0.05) between states when using the non-dominant leg.

**Table 3:** Descriptive statistics for Star Excursion Balance Test performances

Parameters	Limb	Rested state	Fatigued state
ANT RD (%)^a^	DL	108.25 ± 12.09*	103.50 ± 10.85
	NDL	108.26 ± 12.14*	103.20 ± 9.61
PM RD (%)^a^	DL	89.79 ± 10.55*	84.93 ± 12.98
	NDL	89.72 ± 10.93	88.80 ± 10.72
PL RD (%)^b,c^	DL	82.63 ± 12.56^†^	78.82 ± 15.97
	NDL	75.91 ± 12.46	77.82 ± 14.20
COM RD (%)^a^	DL	91.94 ± 10.91	89.08 ± 12.12
	NDL	91.30 ± 10.79	89.94 ± 9.92

ANT anterior, *PM* posteromedial, *PL* posterolateral, *COM* composite, *RD* reach distance, *DL* dominant leg, *NDL* non-dominant leg

*Significant difference between rested and fatigued state

^a^Main effect of state

^b^Main effect of limb

^c^Significant interaction between limb dominance and state

^†^Significant difference between the dominant and non-dominant leg

### Muscle flexibility

Descriptive statistics for the performances in muscle flexibility tests are presented in Table 4. No significant difference was found.

**Table 4:** Descriptive statistics for flexibility test performances

Parameters	Limb	Rested state	Fatigued state
Hamstring (°)	DL	67.77 ± 15.58	64.38 ± 21.26
	NDL	68.85 ± 14.67	66.31 ± 18.83
Gastrocnemius (°)	DL	45.54 ± 8.89	48.46 ± 7.47
	NDL	46.62 ± 9.13	47.92 ± 7.94

*DL* dominant leg, *NDL* non-dominant leg

### Inter-limb asymmetry and fatigue rate of each leg

The magnitude of inter-limb asymmetry for each measurement at the rested and fatigued state, and the fatigue rate of each leg in each measurement are shown in Table 5. The inter-limb asymmetry in triple-hop distance significantly increased at the fatigued state compared to that at the rested state (*Z* = − 1.992, *p* = 0.046, ES = 0.552). The inter-limb asymmetry in ANT reach distance significantly decreased at the fatigued state compared to that at the rested state (*p* = 0.004, ES = 0.993). As for the fatigue rate, the PL reach distance showed a significantly (*p* = 0.028, ES = 0.695) greater decrease, and the COM reach distance showed a tendency (*p* = 0.056) of greater decrease post fatigue when establishing the unilateral stance using the dominant leg compared with using the non-dominant leg.

**Table 5:** Variation of performance post fatigue (fatigue rate) and inter-limb asymmetry

	Limb	Fatigue rate (%)	State	Asymmetry (%)
**Power**				
CMJ	DL	11.56 ± 9.33	Rested	8.20 ± 11.97
	NDL	12.79 ± 9.64	Fatigued	12.76 ± 9.48
Hop	DL	8.45 ± 4.52	Rested	6.64 ± 5.99
	NDL	6.91 ± 5.95	Fatigued	9.59 ± 4.79
Triple Hop	DL	6.17 ± 4.82	Rested	5.78 ± 6.41*
	NDL	8.36 ± 6.35	Fatigued	9.69 ± 6.45
**SEBT**				
ANT RD	DL	4.40 ± 6.95	Rested	8.36 ± 4.75*
	NDL	4.61 ± 7.36	Fatigued	3.71 ± 3.30
PM RD	DL	5.96 ± 8.17	Rested	7.44 ± 5.83
	NDL	1.02 ± 6.95	Fatigued	10.67 ± 4.53
PL RD	DL	5.58 ± 9.62^†^	Rested	9.97 ± 7.93
	NDL	− 2.17 ± 8.04	Fatigued	8.64 ± 6.18
COM RD	DL	5.13 ± 4.51	Rested	6.06 ± 4.08
	NDL	1.42 ± 3.71	Fatigued	4.03 ± 2.17
**Flexibility**				
Hamstring	DL	7.48 ± 16.84	Rested	8.32 ± 6.03
	NDL	5.21 ± 19.18	Fatigued	11.33 ± 8.62
Gastrocnemius	DL	− 6.92 ± 14.92	Rested	7.85 ± 5.45
	NDL	− 3.12 ± 8.30	Fatigued	7.09 ± 6.25

*CMJ* single-leg countermovement jump, *DL* dominant leg, *NDL* non-dominant leg, *ANT* anterior, *PM* posteromedial, *PL* posterolateral, *COM* composite, *RD* reach distance

*Significant difference between the rested and fatigued state

^†^Significant difference between the dominant and non-dominant leg

## Discussion

### Inter-limb asymmetry in lower-limb power

We have reported inter-limb asymmetry in single-leg jump performances among child fencing and Taekwondo athletes (9–11 years old) in our previous study [34]. In the present study, no significant difference was found between limbs at the rested or fatigued state for performance in each jumping test based on group means. However, by calculating the inter-limb asymmetry for each participant, we found that the asymmetry magnitude of the participants was 8.20%, 6.64%, and 5.78% at the rested state, and 12.76%, 9.59%, and 9.69% at the fatigued state for the unilateral CMJ height, hop distance, and triple-hop distance, respectively (Table 5). This finding implies the importance of assessing and quantifying the inter-limb asymmetry in unilateral jump performance on an individual basis. In fact, the inter-limb asymmetry has been shown with a variable nature as the SD was usually close to even higher than the mean [9], which supports the individual approach when taking inter-limb asymmetry as a measurement in practical application. The asymmetry magnitude in the present study was similar with those reported in previous research using the same method of calculation to quantify asymmetry: a 9.9 to 16.8% inter-limb asymmetry in peak force and power in bilateral CMJ was reported in 95% of the collegiate athletes [35], and a 6.3% inter-limb asymmetry in running single-leg jump height was reported in collegiate male basketball athletes [30]. Age-appropriate comparison in asymmetry magnitude is not available due to the paucity of research focused on children.

Regarding the acute effect of fatigue on lower-limb power, our results showed a main effect of state (rested vs. fatigued state) for leg power (single-leg CMJ height, hop distance, and triple-hop distance). The jump performance decreased post fatigue in both legs in all three tests (Table 2), indicating that our protocol was appropriate to induce fatigue. By comparing the magnitude of inter-limb asymmetry between the rested and fatigued state for each test, we found that the asymmetry in triple-hop distance increased post fatigue (Table 5). Most of previous studies have reported similar findings: the inter-limb asymmetry in unilateral CMJ height increased post fatigue among active male adults (aged 28.9 ± 5.1 years) [9] and elite adolescent male soccer athletes (aged 17.6 ± 0.5 years) [15]; the inter-limb asymmetry in peak force, peak power, and mean power during the unilateral CMJ increased post fatigue among male Judo athletes (aged 22.5 ± 3.6 years) [16]. Collectively, these findings indicate that fatigue amplifies the inter-limb asymmetry in leg power, suggesting the necessity of assessing the inter-limb asymmetry at both the non-fatigued and fatigued state. However, based on our results, the conclusion remains elusive regarding the mechanism of the increased asymmetry in triple-hop distance post fatigue as no significant difference in fatigue rate (the variation of performance from rested to fatigued state) was found between legs (Table 5). Jacques et al. [14] reported that the soleus activation amplitude reduced with fatigue in the dominant leg while not in non-dominant leg when examining the muscle activities using electromyography (EMG) during the running movement, suggesting a higher fatigue rate in the dominant leg. More studies are needed to compare the fatigue rate between limbs when examining the acute impact of fatigue on inter-limb asymmetry in leg power as direct evidence is still lacking in current literature. Figuring out this problem may help the athletic trainers developing fatigue-resistant program based on the fatigue response of each leg to improve sport performance and injury prevention for athletes.

### Inter-limb asymmetry in dynamic balance

Postural-control assessments, which can be grouped into static and dynamic balance tests, have been widely used for evaluating risk of injury in sport activities [36, 37]. The underlying task of maintaining standing still in static balance tests may not translate necessarily to sport-movement tasks, and consequently, the performance in static balance tests may not be translated to identifying injury risk for athletes [36, 37]. Although the dynamic balance tests do not exactly replicate the movements in sports, they mimic the demands of sport movements more closely compared with the static balance tests [36]. The SEBT offers a simple, reliable, and low-cost method for assessing dynamic balance and the inter-limb asymmetry in dynamic balance [38] and has been used for pre-pubertal male Taekwondo athletes in previous study [39]. It has been reported that an inter-limb asymmetry greater than 4 cm in ANT reach distance in SEBT indicated increased injury risk among high-school [6] and collegiate athletes [7]. However, it has been suggested that the reach distance should be normalized to leg length in order to accurately compare the performances between participants [40]. We have reported an inter-limb asymmetry ranged from 8.92 to 13.98% in PL reach distance (normalized to leg length) in SEBT among 9–11-year-old male and female fencers and Taekwondo athletes in our previous research [34]. The present study demonstrated similar findings: the 9–11-year-old elite male Taekwondo athletes showed a significant bilateral difference (Table 3) in PL reach distance in SEBT at the rested state (9.97% asymmetry, Table 5). Future research needs to examine the association between injury risk and inter-limb asymmetry in SEBT among child athletes, due to the lack of research focused on children.

Fatigue has been found to decrease reach distances in SEBT [41], whereas there is a lack of research designed to examine the acute impact of fatigue on inter-limb asymmetry in SEBT performance. The present study showed a significant interaction between limb (dominant vs. non-dominant) and state (rested vs. fatigued) for PL reach distance, wherein the significant bilateral difference was only shown at the rested state (Table 3), implying that fatigue rate might differ between the dominant and non-dominant leg. The greater decrement in PL reach distance and a tendency of greater decrement in COM reach distance post fatigue when using the dominant vs. the non-dominant leg for support (Table 5) indicated that the SEBT performance decreased more when using the dominant leg for support, implying a reduced ability of neuromuscular control at the fatigued state when establishing a unilateral stance using the dominant leg. Furthermore, the PM reach distance significantly decreased post fatigue only when using the dominant leg for support (Table 3), which also supported this view. Previous findings have indicated that the reach distances in SEBT were associated with the kinematics [42] and kinetics [43, 44] of the supporting leg. Therefore, the greater decrement of reach distance post fatigue when using the dominant leg for support may indicate a higher fatigue rate in the dominant leg. However, it should be noted that the SEBT challenges comprehensive physiological properties including strength, flexibility, proprioception, and balance [45], and thus, the mechanism of the decreased reach distance in SEBT could be complex. Previous study reported that the specific postural adaptions caused by Taekwondo training were more prominent for the non-dominant leg compared with the dominant leg due to the fact that Taekwondo kicks are more frequently performed using the non-dominant leg for support [39], which might partly explain the mechanism of the higher fatigue rate in the dominant leg in our participants. We suggest future research examine the activity level in lower-limb muscles using EMG during SEBT, and further explore which leg fatigues more by comparing the variation in muscle-activity levels caused by fatigue when using the dominant vs. non-dominant leg for support, since previous research using EMG for SEBT only focused on the dominant leg. This will help generating a better understanding for the influence of fatigue on inter-limb asymmetry in SEBT performance.

By quantifying the inter-limb asymmetry, we found that the asymmetry in ANT reach distance significantly decreased post fatigue (Table 5), which is conflicting with the results in triple-hop tests wherein an increment in asymmetry was shown post fatigue. Whilst challenging to explain, it must be acknowledged that the kinetic and kinematic mechanism is different between the jump tests and SEBT as the latter challenges more comprehensive abilities [45]; therefore, it may not be strange that the variation in inter-limb asymmetry caused by fatigue is different in direction between the two tests. Additionally, although we concluded that the fatigue rate might be greater in the dominant leg, no significant difference was found between legs in the decrease in ANT reach distance post fatigue and the means were close (4.40% vs. 4.61%, Table 5), and thus, the mechanism of the decreased asymmetry in ANT reach distance is unclear. Nevertheless, our findings indicate that the unilateral jump tests and SEBT should not be used interchangeably when assessing the influence of fatigue on inter-limb asymmetry. Another finding of the present study is that the variations in inter-limb asymmetry post fatigue were inconsistent across the reach directions in SEBT. The muscle activation and kinematic strategy have been reported substantially different across the reach directions in SEBT [36], which may explain the inconsistent results between reach directions.

An interesting result is that the PL reach distance increased by 2.17% post fatigue in the non-dominant leg (Table 5). Similar results were reported by Armstrong et al. [42] when examining the effect of fatigue on SEBT performance among university dancers. According to the authors, the increased reach distance might be related to the distinct characteristics of the dance sport as the dancers may have distinct and variable kinematic strategies to maintain or facilitate the SEBT performance under the fatigued condition [42]. In the present study, although the PL reach distance increased with fatigue (2.17%) in the non-dominant leg, this data should be used with caution in practice, due to that the SD (8.04%) was much higher than the mean. Furthermore, this result reflects the variable nature of the inter-limb asymmetry, suggesting the importance of assessment on an individual basis when taking the inter-limb asymmetry in SEBT performance as a measurement in practical application.

### Inter-limb asymmetry in muscle flexibility

Poor flexibility of hamstring and gastrocnemius muscles has been associated with increased risks of lower-limb injuries [26, 46, 47]. Few research from current literature is available on the association between inter-limb asymmetry in muscle flexibility and injury risk. Knapik et al. [5] have reported that female collegiate athletes with a 15% or more bilateral asymmetry in hip extensor sustained more lower-limb injuries. Although this relationship has not been examined on children, practitioners may need to monitor the inter-limb asymmetry in muscle flexibility for child athletes to prevent its potential impact on injury risk. In the present study, the inter-limb asymmetry ranged from 7.09 to 11.33% in hamstring and gastrocnemius muscles at the rested and fatigued state (Table 5). Regarding the impact of fatigue on inter-limb asymmetry in muscle flexibility, there is a paucity of research available from current literature. Our results showed that there was no significant limb by state interaction for hamstring or gastrocnemius flexibility, and no main effect of state was shown (Table 3), implying that the hamstring and gastrocnemius flexibility may not be impacted by fatigue.

### Limitations and recommendations

Although participants ran continuously between trials and no rest intervals were permitted between testing at the fatigued state, one cannot rule out that some level of recovery occurred during the SEBT. Therefore, the fatigued state during the muscle flexibility test might be compromised. For the purpose of time, we only examined hamstring and gastrocnemius flexibility; therefore, we are unable to provide a global quality of lower-limb muscle flexibility. In addition, the sample size is limited as only 13 participants were included in the present study. Future research with a larger sample size is warranted to further examine the effect of fatigue on inter-limb asymmetry in functional performances.

Data of previous injury was not collected, and thus, if previous injury would impact the inter-limb asymmetry in functional performances and the fatigue response is not clear. Previous research has reported that young soccer players (mean age = 11.2 years) showed notably larger inter-limb asymmetry in lateral hop performance compared to that of the younger players (mean age = 9.1 years) [48]. As participants in the present study aged between 9 and 11 years, there might be an effect of age on the inter-limb asymmetry in jump performances. However, whether this would have an impact on our results regarding the effect of fatigue on inter-limb asymmetry in functional performances could not be determined in the present study. In addition, the association between inter-limb asymmetry in functional performances and anthropometric factors (height, weight) has not been reported, and thus, if these factors would impact our results was also unknown. Practitioners also need to be cautious about the skill level (competitive performers) and sex when utilizing the current findings in youth sport training as participants in the present study were elite male child athletes. Future research may consider including these factors (previous injury, age, height, weight, skill level, and sex) as confounders when examining the impact of fatigue on inter-limb asymmetry in functional performances. Findings would also need to be extended from Taekwondo to other sports.

We mainly discussed inter-limb asymmetry in functional performances in the present study, while the absolute performance was not discussed. When utilizing the measurement of inter-limb asymmetry in functional performances for injury prevention, practitioners also need to pay attention to the absolute performance of each side as poor performance on both limbs will be categorized as symmetrical. Further, it is interesting to consider if great functional performance could compensate the inter-limb asymmetry, and the potential effect of this on injury risk.

## Conclusions

Fatigue significantly impacts inter-limb asymmetry in jump performances and dynamic balance in child athletes. The variation of inter-limb asymmetry caused by fatigue may be different across tests. Fatigue rates differ between the dominant and non-dominant leg, while further research needs to explore which leg fatigues more. For the purpose of injury prevention, practitioners should consider assessing the inter-limb asymmetry at both the rested and fatigued state for children and be mindful of the fatigue response of each leg in functional tests.

Future research should explore the potential factors (e.g., limb dominance and leg length discrepancy) associated with the discrepancy of fatigue rate between limbs. We suggest using EMG to examine the activity level of lower-limb muscles in functional tests, and further explore which leg fatigues more by comparing the variation of muscle-activity levels post fatigue. In addition, future research may consider comparing the present study with numerical simulation and measuring this fatigue with finite element analysis.

## Data Availability

The datasets used and/or analyzed during the current study are available from the corresponding author on reasonable request.

## Abbreviations

ANT: Anterior
CMJ: Countermovement jump
COM: Composite
ES: Effect size
ICC: Intra-class correlation coefficient
PL: Posterolateral
PM: Posteromedial
SD: Standard deviation
SEBT: Star Excursion Balance Test
